# Mitochondrial Unfolded Protein Response to Microgravity Stress in Nematode *Caenorhabditis elegans*

**DOI:** 10.1038/s41598-019-53004-9

**Published:** 2019-11-11

**Authors:** Peidang Liu, Dan Li, Wenjie Li, Dayong Wang

**Affiliations:** Medical School, Southeast University, Nanjing, 210009 China

**Keywords:** Environmental impact, Risk factors

## Abstract

*Caenorhabditis elegans* is useful for assessing biological effects of spaceflight and simulated microgravity. The molecular response of organisms to simulated microgravity is still largely unclear. Mitochondrial unfolded protein response (mt UPR) mediates a protective response against toxicity from environmental exposure in nematodes. Using HSP-6 and HSP-60 as markers of mt UPR, we observed a significant activation of mt UPR in simulated microgravity exposed nematodes. The increase in HSP-6 and HSP-60 expression mediated a protective response against toxicity of simulated microgravity. In simulated microgravity treated nematodes, mitochondria-localized ATP-binding cassette protein HAF-1 and homeodomain-containing transcriptional factor DVE-1 regulated the mt UPR activation. In the intestine, a signaling cascade of HAF-1/DVE-1-HSP-6/60 was required for control of toxicity of simulated microgravity. Therefore, our data suggested the important role of mt UPR activation against the toxicity of simulated microgravity in organisms.

## Introduction

During the spaceflight, the significant risk on movement, muscle, and metabolism of human beings and animals have been frequently observed^1–5^. Microgravity contributes to the detected pathological alterations during spaceflight^1,4^. Simulated microgravity treatment is an important strategy to predict the possible toxicity of microgravity and to elucidate the underlying mechanisms. The simulated microgravity can also result in the abnormal psychological performance, endocrine, and intestinal dysfunction as observed by microgravity during the spaceflight^6–9^.

Nematode *Caenorhabditis elegans*, a classic model animal, is a wonderful model for toxicological study of stresses or toxicants^10–14^. *C*. *elegans* is a suitable model for assessing effects of microgravity^15,16^. With the work in “the first International *C*. *elegans* Experiment in Space” (ICE-First) experiments as an example, it has been observed that microgravity could potentially at least cause the toxicity on early embryogenesis, muscle development, germline development, locomotion behavior, and reproduction in nematodes^17–23^. The toxicity of simulated microgravity on nematodes could be further assessed more recently^24–27^. The observed toxicity induced by simulated microgravity was under the control of insulin and p38 mitogen-activated protein kinase (MAPK) signaling pathways in nematodes^24,26^.

In the mitochondrion, mitochondrial unfolded protein response (mt UPR) mediates a protective response against the toxicity from environmental stresses or toxicants in nematodes^28^. However, the response of mt UPR signaling to simulated microgravity remains largely unclear. We here examined the induction of mt UPR in simulated microgravity treated nematodes and the underlying mechanism. Our data demonstrated the noticeable activation of mt UPR in simulated microgravity treated nematodes. Moreover, the mtUPR signaling was involved in the regulation of response to simulated microgravity.

## Results

### Simulated microgravity induced the mt UPR in nematodes

**Figure 1:**
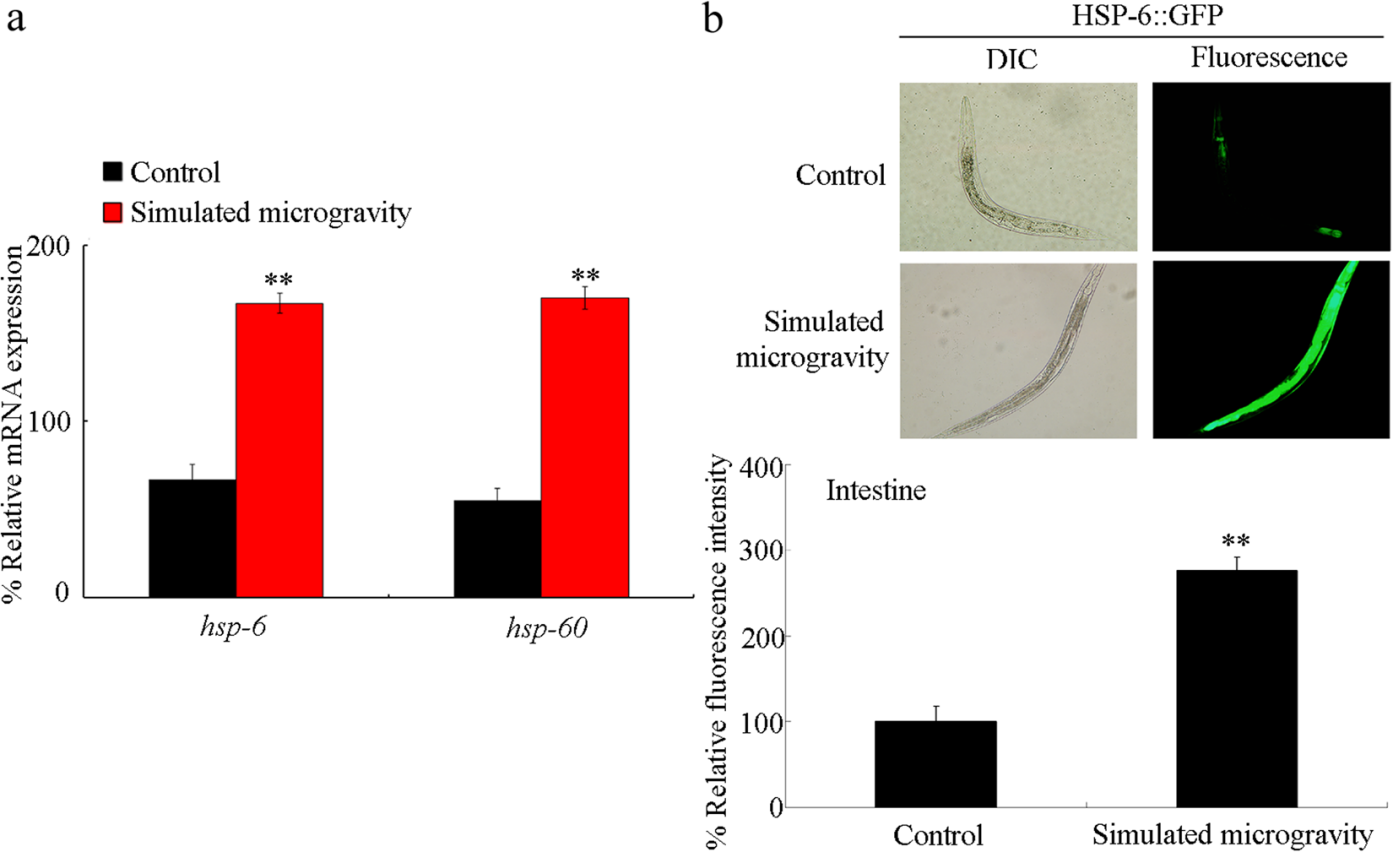
Simulated microgravity treatment induced the mt UPR in nematodes. (**a**) Effect of simulated microgravity treatment on expression of *hsp-6* and *hsp-60*. Relative expression ratio between the examined genes and the reference gene (*tba-1*) was determined. (**b**) Effect of simulated microgravity treatment on expression of intestinal HSP-6::GFP. DIC, differential interference contrast. Simulated microgravity treatment was performed for 24-h. Bars represent means ± SD. ^**^P < 0.01 *vs* Control.

In nematodes, HSP-60 and HSP-6 are mt UPR markers^29,30^. Simulated microgravity treatment (24-h) caused a significant increase in expression of both *hsp-6* and *hsp-60* (Fig. 1a). HSP-6 can be expressed in intestinal cells^31^. Meanwhile, using the transgenic strain of *zcIs13*[HSP-6::GFP], we found an obvious increase in HSP-6::GFP expression in the intestine of nematodes treated with simulated microgravity (Fig. 1b).

### Effect of RNAi knockdown of *hsp-6* or *hsp-60* on toxicity of simulated microgravity

**Figure 2:**
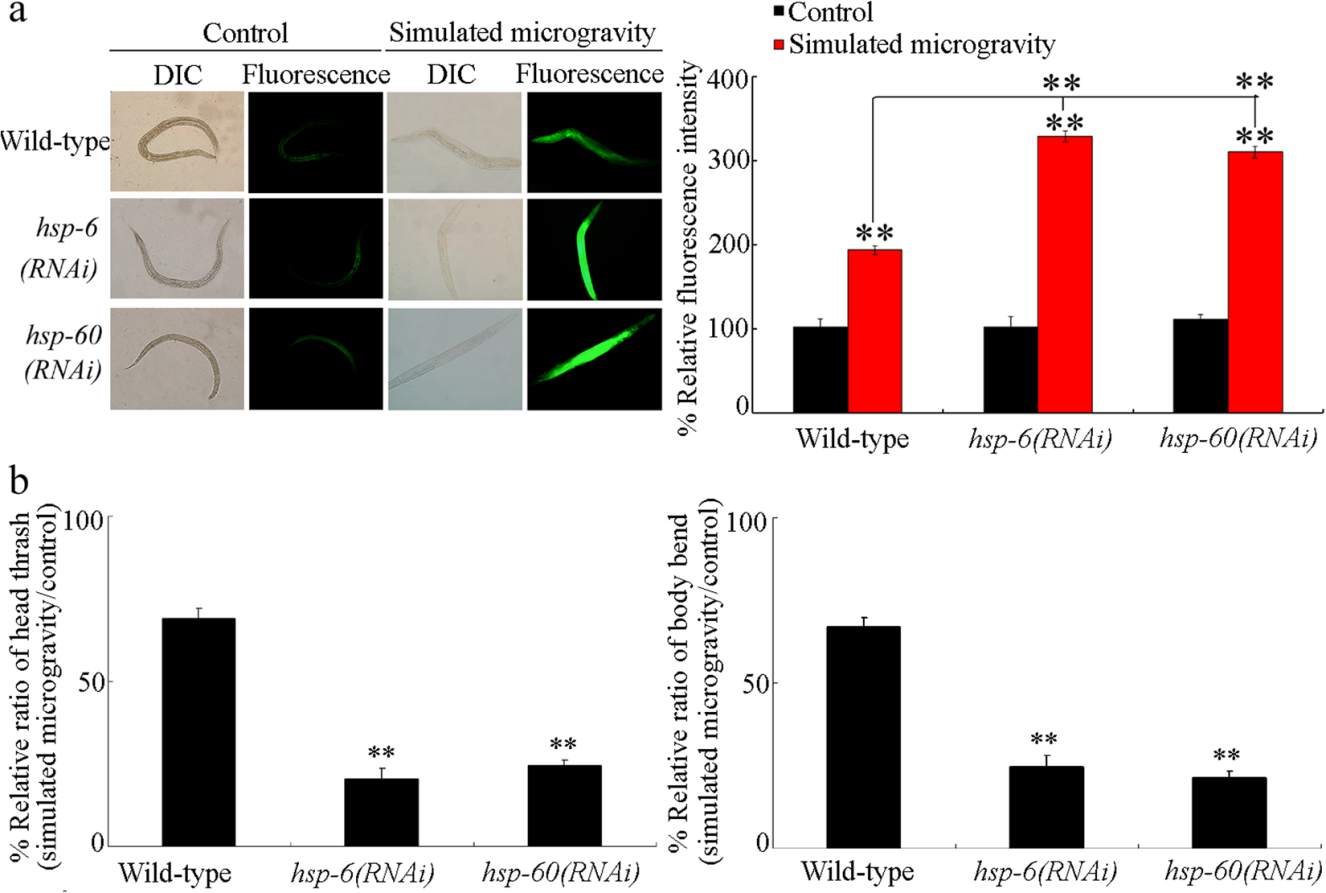
RNAi knockdown of *hsp-6* or *hsp-60* induced a susceptibility to the toxicity of simulated microgravity. (**a**) RNAi knockdown of *hsp-6* or *hsp-60* caused a susceptibility to the toxicity of simulated microgravity in inducing intestinal ROS production. DIC, differential interference contrast. Bars represent means ± SD. ^**^P < 0.01 *vs* Control (if not specially indicated). (**b**) RNAi knockdown of *hsp-6* or *hsp-60* caused a susceptibility to the toxicity of simulated microgravity in decreasing locomotion behavior. Considering that the examined nematodes have deficit in locomotion behavior, the locomotion behavior was expressed as the ratio between simulated microgravity and control. Bars represent means ± SD. ^**^P < 0.01 *vs* Wild-type. Simulated microgravity treatment was performed for 24-h.

We detected the more severe induction of intestinal reactive oxygen species (ROS) production and decrease in locomotion behavior in simulated microgravity treated *hsp-6(RNAi)* or *hsp-60(RNAi)* nematodes compared with those in simulated microgravity treated wild-type nematodes (Fig. 2). Thus, RNAi knockdown of *hsp-6* or *hsp-60* induced a susceptibility to toxicity of simulated microgravity.

### HAF-1 and DVE-1 were involved in the regulation of response to simulated microgravity

**Figure 3:**
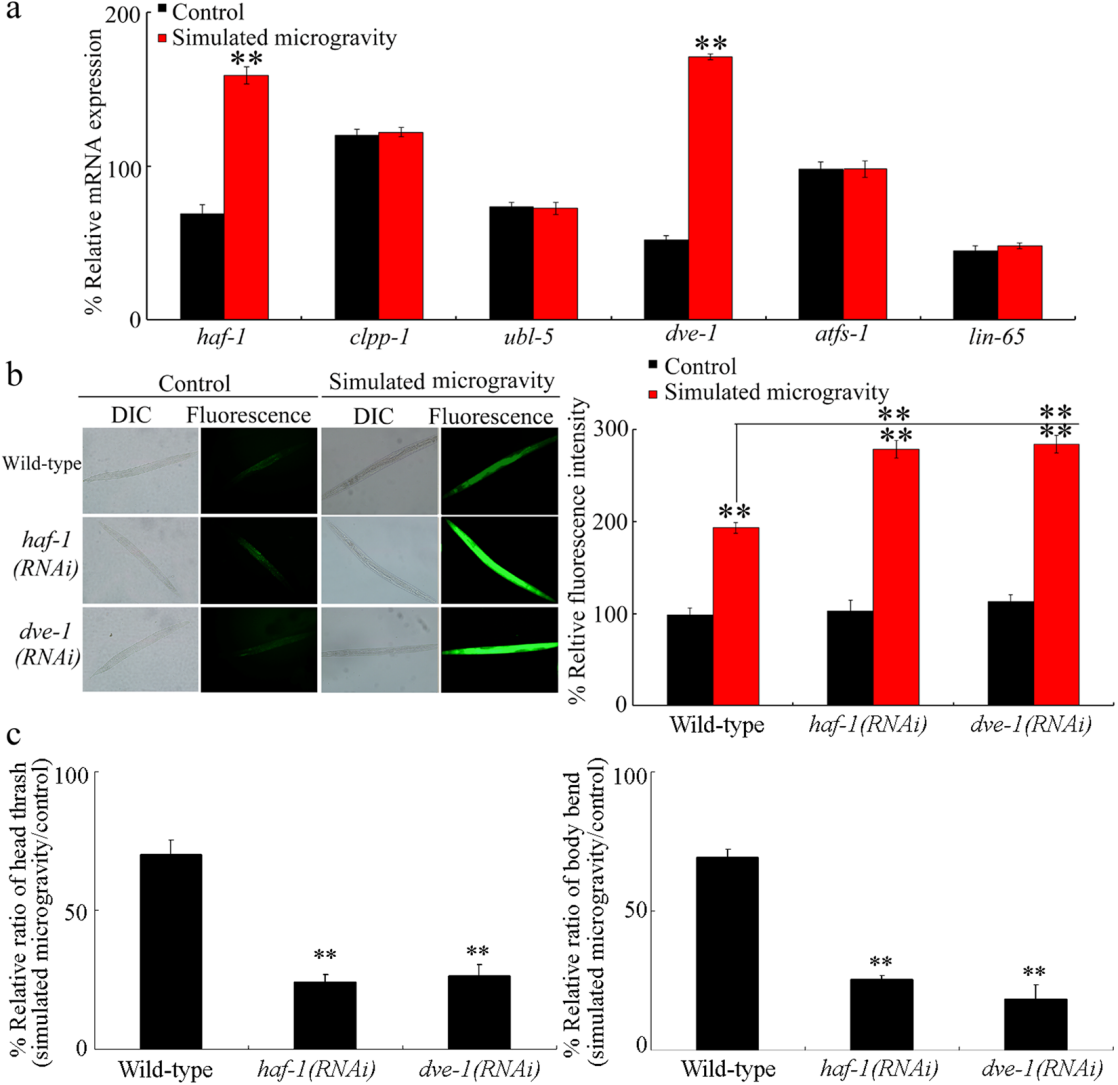
HAF-1 and DVE-1 were involved in the control of response to simulated microgravity. (**a**) Effect of simulated microgravity on expressions of *haf-1*, *clpp-1*, *ubl-5*, *dev-1*, *atfs-1*, and *lin-65*. Relative expression ratio between the examined genes and the reference gene (*tba-1*) was determined. Bars represent means ± SD. ^**^P < 0.01 *vs* Control. (**b**) RNAi knockdown of *haf-1* or *dve-1* induced a susceptibility to toxicity of simulated microgravity in inducing intestinal ROS production. DIC, differential interference contrast. Bars represent means ± SD. ^**^P < 0.01 *vs* Control (if not specially indicated). (**c**) RNAi knockdown of *haf-1* or *dve-1* induced a susceptibility to toxicity of simulated microgravity in decreasing locomotion behavior. Considering that the examined nematodes have deficit in locomotion behavior, the locomotion behavior was expressed as the ratio between simulated microgravity and control. Bars represent means ± SD. ^**^P < 0.01 *vs* Wild-type^.^ Simulated microgravity treatment was performed for 24-h.

In nematodes, some proteins, such as ATFS-1, DVE-1, UBL-5, HAF-1, CLPP-1, and LIN-65, are required for mt UPR induction by regulating the expression of mt UPR markers during the stress response^32–35^. Simulated microgravity treatment (24-h) caused a significant increase in expressions of *haf-1* and *dve-1* (Fig. 3a). We did not observe the significant alteration in expression of other genes in simulated microgravity treated nematodes (Fig. 3b).

We further found that RNAi knockdown of *haf-1* or *dve-1* induced the more severe toxicity of simulated microgravity in inducing intestinal ROS production and in decreasing locomotion behavior compared with those in wild-type nematodes (Fig. 3b,c), suggesting that the nematodes with RNAi knockdown of *haf-1* or *dve-1* were susceptible to the toxicity of simulated microgravity.

### Intestine-specific activity of HAF-1, DVE-1, HSP-6, or HSP-60 in regulating the response to simulated microgravity

**Figure 4:**
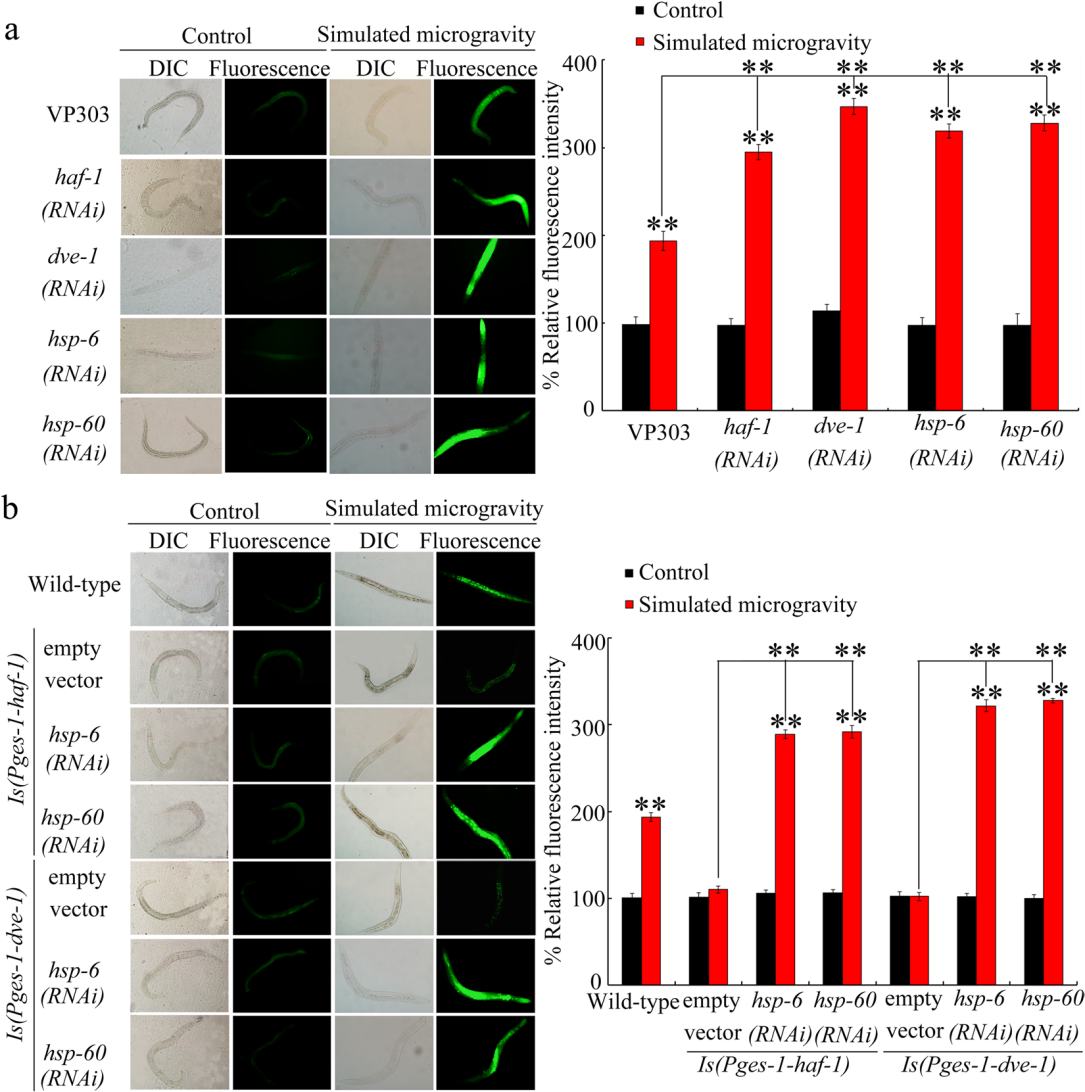
Intestine-specific activity of mt UPR related genes in regulating the toxicity of simulated microgravity. (**a**) Effect of RNAi knockdown of *haf-1*, *dev-1*, *hsp-6*, or *hsp-60* on toxicity of simulated microgravity in inducing intestinal ROS production. (**b**) Effect of RNAi knockdown of *hsp-6* or *hsp-60* on intestinal ROS production in simulated microgravity treated nematodes with intestinal overexpression of HAF-1 or DVE-1. DIC, differential interference contrast. Empty vector, L4440. Bars represent means ± SD. ^**^P < 0.01 *vs* Control (if not specially indicated). Simulated microgravity treatment was performed for 24-h.

In nematodes, intestinal insulin and p38 MAPK signaling pathways play a crucial function in regulating the response to simulated microgravity^24,26^. We next focused on the intestine to determine the activity of HAF-1, DEV-1, HSP-6, and HSP-60 in regulating simulated microgravity toxicity. Simulated microgravity treatment significantly increased the expressions of *haf-1*, *dve-1*, *hsp-6*, and *hsp-60* in isolated intestines (Fig. S1). After the simulated microgravity treatment, intestine-specific RNAi knockdown of *haf-1*, *dev-1*, *hsp-6*, or *hsp-60* resulted in a more severe intestinal ROS production compared with VP303 nematodes (Fig. 4a), suggesting the susceptibility of nematodes with intestine-specific RNAi knockdown of *haf-1*, *dev-1*, *hsp-6*, or *hsp-60* to simulated microgravity toxicity. Since the VP303 nematodes has defect in locomotion behavior, we did not further investigate locomotion behavior phenotypes.

### Genetic interaction of HSP-6/60 with HAF-1 or DVE-1 in regulating the response to simulated microgravity

To determine the interaction of HSP-6/HSP-60 with HAF-1 or DEV-1 in regulating the response to simulated microgravity, transgenic strain overexpressing intestinal HAF-1 or DEV-1 was generated. Under the condition without the simulated microgravity treatment, the nematodes overexpressing intestinal HAF-1 or DEV-1 do not show obvious intestinal ROS production (Fig. 4b). Intestinal overexpression of HAF-1 or DEV-1 suppressed the toxicity of simulated microgravity in inducing intestinal ROS production, demonstrating the resistance of nematodes overexpressing intestinal HAF-1 or DEV-1 to simulated microgravity toxicity (Fig. 4b). Moreover, RNAi knockdown of *hsp-6* or *hsp-60* effectively inhibited the resistance of nematodes overexpressing intestinal HAF-1 or DEV-1 to the toxicity of simulated microgravity in inducing intestinal ROS production (Fig. 4b). Therefore, HSP-6/60 acted downstream of intestinal HAF-1 or DEV-1 to regulate the response to simulated microgravity.

### HAF-1 and DVE-1 regulated the mt UPR activation in simulated microgravity treated nematodes

**Figure 5:**
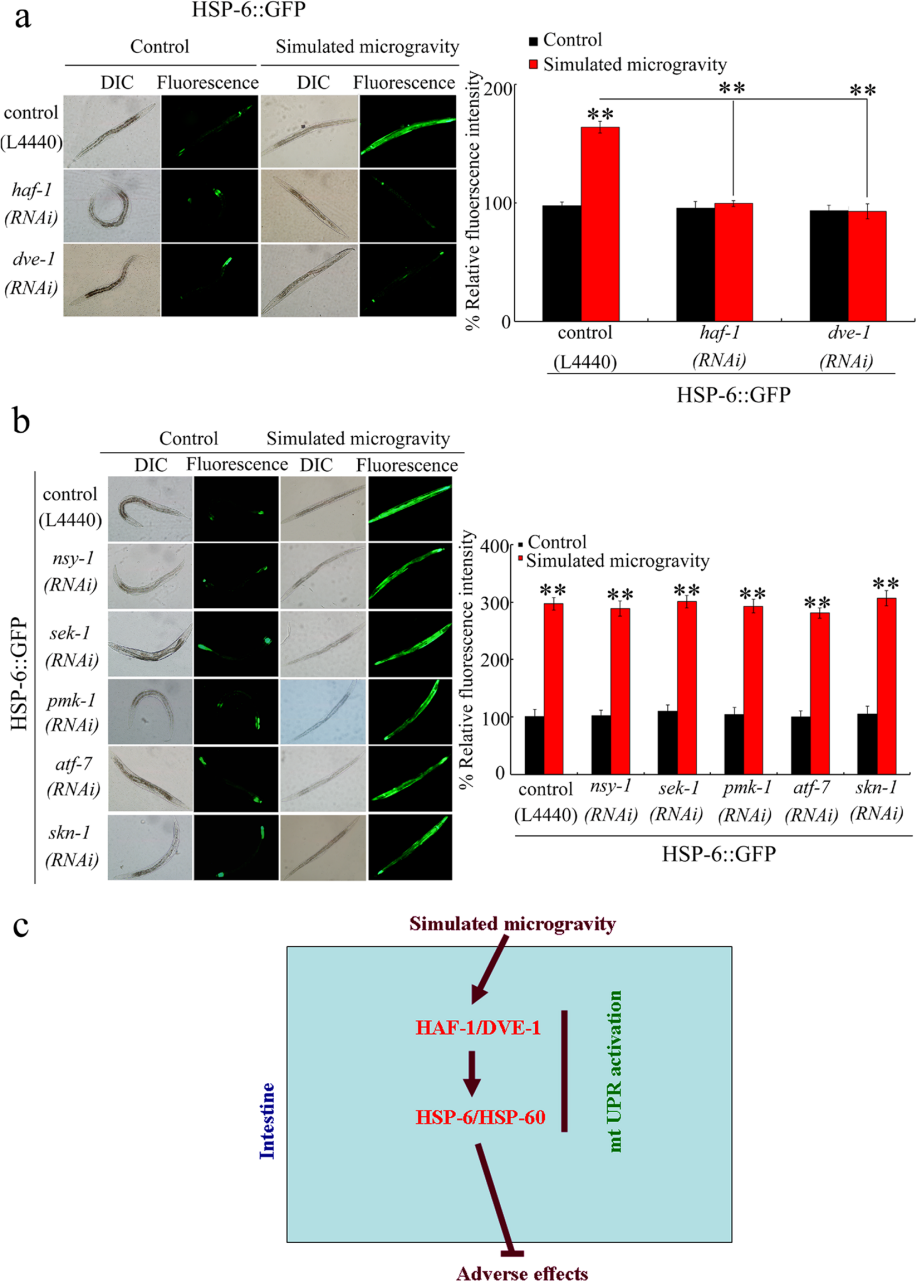
RNAi knockdown of *haf-1* or *dev-1* regulated the mt UPR in simulated microgravity treated nematodes. (**a**) Effect of RNAi knockdown of *haf-1* or *dev-1* on HSP-6::GFP induction in simulated microgravity treated nematodes. (**b**) Effect of RNAi knockdown of *nsy-1*, *sek-1*, *pmk-1*, *atf-7*, or *skn-1* on HSP-6::GFP induction in simulated microgravity treated nematodes. DIC, differential interference contrast. Empty vector, L4440. Simulated microgravity treatment was performed for 24-h. Bars represent means ± SD. ^**^P < 0.01 *vs* Control (if not specially indicated). (**c**) A diagram showing the role of mt UPR signaling pathway in regulating the response to simulated microgravity in nematodes.

To confirm the function of HAF-1 and DVE-1 in modulating mt UPR activation in simulated microgravity treated nematodes, we carried our RNAi knockdown of *haf-1* or *dve-1* in *zcIs13*[HSP-6::GFP] nematodes. RNAi knockdown of *haf-1* or *dve-1* obviously suppressed the activation of HSP-6::GFP induced by simulated microgravity (Fig. 5a), suggesting the requirement of HAF-1 and DVE-1 for the mt UPR activation in simulated microgravity treated nematodes.

### p38 MAPK signaling pathway was not involved in the regulation of mt UPR activation in simulated microgravity treated nematodes

Our previous study has raised an intestinal cascade of NSY-1-SEK-1-PMK-1-ATF-7/SKN-1 required for the control of simulated microgravity toxicity in nematodes^24^. Nevertheless, we found that RNAi knockdown of *nsy-1*, *sek-1*, *pmk-1*, *atf-7*, or *skn-1* in the p38 MAPK signaling pathway did not obviously influence HSP-6::GFP induction in simulated microgravity treated nematodes (Fig. 5b), which suggests that the p38 MAPK signaling pathway did not regulate the mt UPR activation induced by simulated microgravity.

### Effect of intestinal RNAi knockdown of *hsp-6* or *hsp-60* on mitochondrial dysfunction, mitochondrial ROS production, and mitophagy in simulated microgravity treated nematodes

We used the oxygen consumption rate and mitochondrial membrane potential to reflect the mitochondrial function, and found that simulated microgravity treatment could significantly decrease the oxygen consumption ratio and reduce the mitochondrial membrane potential (Fig. S2a,b). Meanwhile, treatment with simulated microgravity induced the significant mitochondrial ROS production (Fig. S2c). We employed *dct-1* and *pink-1* as molecular markers of mitophagy in nematodes^36^. Simulated microgravity treatment induced the significant increase in expression of both *dct-1* and *pink-1* in nematodes (Fig. S2d).

Moreover, we observed that intestinal RNAi knockdown of *hsp-6* or *hsp-60* induced the more severe decrease in oxygen consumption ratio and reduction in mitochondrial membrane potential compared with VP303 nematodes after simulated microgravity treatment (Fig. S2a,b). In simulated microgravity treated VP303 nematodes, intestinal RNAi knockdown of *hsp-6* or *hsp-60* caused the more severe induction of mitochondrial ROS production (Fig. S2c). Furthermore, intestinal RNAi knockdown of *hsp-6* or *hsp-60* significantly suppressed the increase in expressions of both *dct-1* and *pink-1* induced by simulated microgravity treatment (Fig. S2d).

## Discussion

Using HSP-6 and HSP-60 as the mt UPR markers, the significant induction in expression of HSP-6 and HSP-60 was observed in simulated microgravity treated nematodes (Fig. 1). This observation demonstrated the potential noticeable induction of mt UPR by simulated microgravity in organisms. Simulated microgravity also induced the mitochondrial dysfunction in rat cerebral arteries^37^. Thus, simulated microgravity may at least activate two different responses in mitochondrion of organisms. One is the mitochondrial dysfunction, and another is the mt UPR activation.

We further found that RNAi knockdown of *hsp-6* or *hsp-60* could induce a susceptibility to simulated microgravity toxicity (Fig. 2), which indicated that the induction of HSP-6 or HSP-60 mediates a protective mt UPR response to simulated microgravity. The microgravity also potentially induced the proteomics changes involved in endoplasmic reticulum (ER) response^38,39^. That is, both mt UPR and ER UPR may be potentially activated by simulated microgravity treatment in organisms.

During the control of mt UPR, mitochondria-localized ATP-binding cassette protein HAF-1 governs export of peptides from matrix, which is required for mt UPR signaling across mitochondrial inner membrane^35^. Mitochondrial matrix protease CLPP-1 mediates the generation of peptides in mitochondrial matrix^34,35^. The mt UPR activation correlates with the nuclear redistribution of transcriptional factor DVE-1, and complex formation between DVE-1 and small ubiquitin-like protein UBL-5^34^. Mitochondrial import efficiency of another transcriptional factor ATFS-1 is also required for mt UPR activation^33^. The mt UPR activation also requires nuclear co-factor LIN-65^32^. Among the genes encoding these proteins, we found that simulated microgravity only affected expressions of *haf-1* and *dve-1* (Fig. 3a). This observation suggested that simulated microgravity may only affect activity of transcriptional factor DVE-1, but not influence the activities of another transcriptional factor ATFS-1 and nuclear co-factor LIN-65. Additionally, in the complex of DVE-1-UBL-5, simulated microgravity may be not able to influence activity of UBL-5. During the activation of mt UPR, simulated microgravity may affect export process of peptides from the matrix controlled by HAF-1, but not influence the CLPP-1-mediated generation of peptides in mitochondrial matrix. HSP-6 is an ortholog of human HSP70, HSP-60 is an ortholog of human HSP60, HAF-1 is an ortholog of human ABCB10, and DVE-1 is an ortholog of human STAB2. It was reported that the simulated microgravity could upregulate the expressions of HSP60 and HSP70 in human bone stem cells^40^.

The functional analysis further confirmed the involvement of HAF-1 and DVE-1 in regulating the response to simulated microgravity (Fig. 3b,c). Our previous studies have suggested that oxidative stress-related, insulin, and p38 MAPK signaling pathways were required for toxicity induction of simulated microgravity in nematodes^24,26,41^. Our data further suggests the involvement of mt UPR signaling pathway in regulating the response to simulated microgravity.

We further provide the evidence to indicate the intestine-specific activity of HAF-1, DEV-1, HSP-6, and HSP-60 in modulating the response to simulated microgravity (Fig. 4a). That is, besides insulin and p38 MAPK signaling pathways, mt UPR signaling also acted in the intestine to regulate the response to simulated microgravity. We further raised an intestinal signaling cascade of HAF-1/DEV-1-HSP-6/60 required for the regulation of response to simulated microgravity (Fig. 4b). Nevertheless, p38 MAPK signaling pathway was not required for the activation of mt UPR induced by simulated microgravity (Fig. 5b). However, RNAi knockdown of *haf-1* or *dve-1* inhibited the activation of mt UPR induced by simulated microgravity (Fig. 5a). Therefore, mt UPR and p38 MAPK signaling may mediate two different molecular responses to simulated microgravity in nematodes.

In this study, we further found that the mt UPR activation was associated with induction of mitochondrial dysfunction, mitochondrial ROS production, and mitophagy in simulated microgravity treated nematodes. After simulated microgravity treatment, we detected the decrease in oxygen consumption ratio (Fig. S2a), the reduction in mitochondrial membrane potential (Fig. S2b), the induction of mitochondrial ROS production (Fig. S2c), and the activation of mitophagy (Fig. S2d). Moreover, our data suggested that the mt UPR signaling may regulate mitochondrial dysfunction, mitochondrial ROS production, and mitophagy in simulated microgravity treated nematodes. We detected the more severe decrease in oxygen consumption ratio, reduction in mitochondrial membrane potential, and induction of mitochondrial ROS production in *hsp-6(RNAi)* or *hsp-60(RNAi)* nematodes compared with VP303 after simulated microgravity treatment (Fig. S2a–c). Additionally, RNAi knodown of *hsp-6* or *hsp-60* suppressed the mitophagy activation induced by simulated microgravity (Fig. S2d).

Together, we examined the mt UPR activation induced by simulated microgravity in nematodes. We detected a significant activation of mt UPR in nematodes treated with simulated microgravity. The increase in expressions of HSP-6 and HSP-60 mediated a protective response to simulated microgravity. In simulated microgravity treated nematodes, HAF-1 and DEV-1 regulated the activation of mt UPR. Moreover, we raised an intestinal signaling cascade of HAF-1/DEV-1-HSP-6/60 involved in the regulation of response to simulated microgravity (Fig. 5c). These findings highlight the crucial protective function of mt UPR activation against the toxicity of simulated microgravity in organisms.

## Methods

### Simulated microgravity

Simulated microgravity was performed as described^26^. Young adults (approximately 100 young adults) were suspended in a soft and movable agar medium (0.2%, half filled) in chamber of Rotary System^TM^ (Synthecon). Balancing sedimentation-induced gravity by centrifugation (horizontally at 30 rpm for 24 h) was carried out to generate the simulated microgravity^42^. Control young adults were maintained in 0.2% agar medium without microgravity treatment.

### Animal maintenance

Transgenic strains (VP303/*kbIs7[nhx-2p::rde-1]* and SJ4100/*zcIs13*[HSP-6::GFP]) and wild-type N2 were used in this study. Animals were maintained normally on nematode growth medium (NGM) plates as described^43^. VP303 is used for intestine-specific RNA interference (RNAi) knockdown of certain gene^44^. NGM plates were fed with food for nematodes, *Escherichia coli* OP50. Bleaching mixture (2% HOCl, 0.45 M NaOH) was used to treat the gravid nematodes in order to collect eggs and to prepare age synchronous L1-larvae or young adults.

### Intestinal ROS production

ROS production was used to reflect the activation of oxidative stress^45^. ROS production was examined as described^46^. Nematodes were labeled using 1 μM CM-H_2_DCFDA for 3 h in the darkness. The nematodes were analyzed for the excitation wavelength at 488 nm and the emission filter at 510 nm under a laser scanning confocal microscope. Relative ROS signal fluorescence intensity was semi-quantified in relative to the total protein concentration. Fifty nematodes were analyzed per treatment.

### Locomotion behavior

Locomotion behaviors were used to reflect the functional state of motor neurons^47^. Locomotion behavior was examined based on two endpoints (head thrash and body bend) as described^48^. A change for bending direction at body mid-region of nematodes has been recorded as a head thrash. The head thrash was analyzed in 1 min. A change of posterior bulb direction has been recorded as a body bend. The body bend was analyzed in 20 sec. Considering that some the nematodes with RNAi knockdown of certain gene (such as *haf-1* or *dve-1*) required for the control of mt UPR have deficit in locomotion behavior, the locomotion behavior was expressed as the ratio between simulated microgravity and control. Forty nematodes were analyzed per treatment.

### Quantitative real-time polymerase chain reaction (qRT-PCR)

The reagent of Trizol (Invitrogen) was used to extract the RNAs. Using ABI 7500 real-time PCR system with Evagreen (Biotium), qRT-PCR was performed to analyze the expression of genes required for the mt UPR activation. Transcriptional expression ratio between genes required for the mt UPR and reference gene (*tba-1*) was determined. Biological reactions were carried out for three times. Table S1 shows the primer information.

### RNAi

The L1-larvae were fed with HT115 (*E*. *coli* strain) carrying double-stranded RNA corresponding to certain gene(s)^49^. Once the L1 larvae on RNAi plates became the gravid animals, they were picked on fresh RNAi plate to lay eggs. The second generation was used for simulated microgravity treatment. HT115 bacteria expressing empty vector L4440 was selected as a negative control. Efficiency of RNAi was confirmed by qRT-PCR (data not shown).

### DNA construction

PCR was performed using genomic DNA to amply intestine-specific *ges-1* promoter. *haf-1* or *dve-1* cDNA fragment was subcloned into pPD_95_77 vector carrying the *ges-1* promoter. Gene transformation was performed by coinjecting 10–40 μg/mL testing DNA and 60 μg/mL marker DNA (*Pdop-1::rfp*) into the gonad^50^. Table S2 shows the related primer information.

### Oxygen consumption rate

The oxygen consumption rate was measured as described^51^. After microgravity treatment, the examined nematodes were washed with M9 buffer for three times. The nematodes were then diluted to 500 worms per 50 ml and incubated in Mitocell chamber. The slopes of linear portions of plots were used to assess the oxygen consumption rates. Three independent trials were performed.

### Mitochondrial membrane potential

Tetramethylrhodamine ethyl ester (TMRE) uptake was used to measure the mitochondrial membrane potential^52^. After microgravity treatment, the examined nematodes were washed with M9 buffer for three times. The nematodes were then labeled with TMRE (0.1 μM) for 24-h. Relative TMRE fluorescence intensity was examined under a laser scanning confocal microscope. Fifty nematodes were analyzed per treatment.

### Mitochondrial ROS production

After microgravity treatment, the examined nematodes were washed with M9 buffer for three times. To determine the mitochondrial ROS production, the nematodes were labeled with 0.5 μM MitoTracker^®^Red CM H2XRos for 48-h^53^. Relative fluorescence intensity was examined under a laser scanning confocal microscope. Fifty nematodes were analyzed per treatment.

### Statistical analysis

SPSS 12.0 software was used for statistical analysis. One-way analysis of variance (ANOVA) was used to analyze the differences between groups. Two-way ANOVA analysis was used for the examination of multiple factor comparison. Probability level of 0.01 (^**^) was considered to be statistically significant.

## Supplementary information

Supporting information
