# Supplementary material for: Mitochondrial Unfolded Protein Response to Microgravity Stress in Nematode Caenorhabditis elegans
Source: Sci Rep. 2019 Nov 11;9:16474. doi: 10.1038/s41598-019-53004-9 (PMC6848112; doi:10.1038/s41598-019-53004-9)
Supplement: Supplementary file 1 — Supporting information [file 41598_2019_53004_MOESM1_ESM.pdf]

Mitochondrial Unfolded Protein Response to Microgravity Stress in Nematode *Caenorhabditis elegans*

Peidang Liu, Dan Li, Wenjie Li & Dayong Wang*

Medical School, Southeast University, Nanjing 210009, China

*Corresponding author.

E-mail address: dayongw@seu.edu.cn (D. Wang).

Supporting Information:

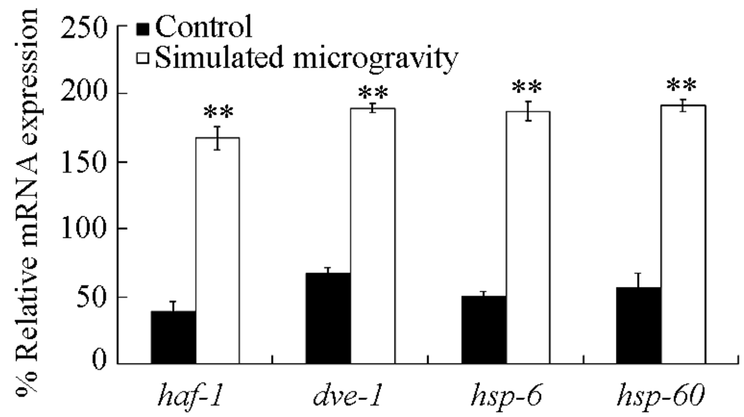

Figure S1. Effect of simulated microgravity treatment on expression of *haf-1*, *dve-1*, *hsp-6* and *hsp-60* in the intestine. The intact intestines were isolated for RNA extraction, and average 30 intact intestines were isolated. Relative expression ratio between the examined genes and the reference gene (*tba-1*) was determined. Simulated microgravity treatment was performed for 24-h. Bars represent means ± SD. $^{**}P < 0.01$ *vs* Control.

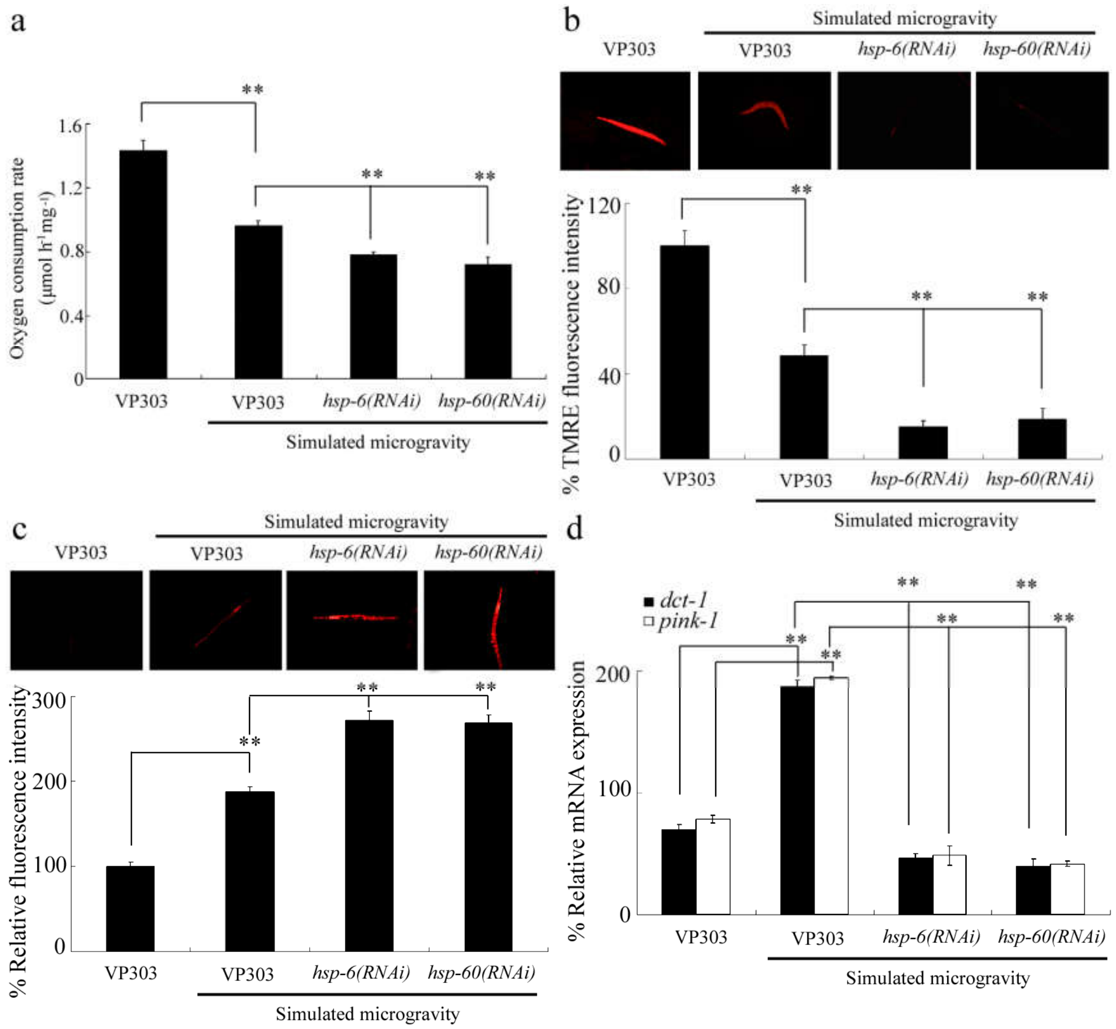

Figure S2. Effect of intestinal RNAi knockdown of *hsp-6* or *hsp-60* on mitochondrial dysfunction, mitochondrial ROS production, and mitophagy in simulated microgravity treated nematodes. (**a**) Oxygen consumption rate. (**b**) Mitochondrial membrane potential. (**c**) Mitochondrial ROS production. (**d**) Mitophagy activation assay as indicated by *dct-1* and *pink-1* expressions. Relative expression ratio between the examined genes and the reference gene (*tba-1*) was determined. Simulated microgravity treatment was performed for 24-h. Bars represent means ± SD. $^{**}P < 0.01$.

Table S1. Primers used for quantitative real-time PCR of genes

Gene	Forward Primer (5'-3')	Reverse Primer (5'-3')
tba-1	TCAACACTGCCATCGCCGCC	TCCAAGCGAGACCAGGCTTCAG
hsp-6	ATTCTTGTCTTCCGCTCGCA	CAGCAGTGAAAGCAACCGTC
hsp-60	CCGTCTCTGTCACTATGGGC	CTCGAATCCCTCTTTGGCGA
haf-1	GTTAATCAGACTGCGATCGAGCG	CGGGACATATCACATATGGAGAGC
clpp-1	AACAGTCCAGGCGGCAGTGT	TTCACCAGCCGAGTGGTGGT
ubl-5	TTGAAATCACAGTAAACGATCGAC	AGCTCGAAATTGAATCCCTCGT
dve-1	CGTCGAAACTGTACTAGCAGC	CGTTTCCAGCGTATCAAGCC
atfs-1	GGTTTGCGGCAGAGATCCTA	AGCTGATCGATCGCCGATTT
lin-65	AGAAGAGCTCCACCATTGGC	TGGTAGACGTGTCGAATCGC
dct-1	CTGGTATGTCAGAATCGTGGGT	CTCTGGCTCGGGAGAGTTTG
pink-1	GCAGCTGGAGGCCTTTCTTA	GCTCGAAGTTGTCGTTCTGC

Table S2. Primer information for DNA constructs

Gene	Forward primer (5'-3')	Reverse primer (5'-3')
Pges-1	ATATCTAGAAGCCACTCAGCCACTTCA	ATAGGATCCCATCTGAATTCAAAGATA
haf-1/C30H6.6.1	ATACCCGGGATGCACTGTCTATGTGTT	GCGGGTACCGAATTTTAATGAAAATCC
dve-1/ZK1193.5a.1	ATAGGATCCATGTTCCCAATGAGGGTA	GTGGGTACCTACGAAAACCTTCTGCTC
